# CoFe_2_O_4_ on Mica Substrate as Flexible Ethanol Gas Sensor in Self-Heating Mode

**DOI:** 10.3390/s24061927

**Published:** 2024-03-17

**Authors:** Jong Hun Kim, Yeong Uk Choi, Jong Hoon Jung, Jae-Hun Kim

**Affiliations:** 1 Department of Physics, Inha University, Incheon 22212, Republic of Korea; jh_kim@inha.ac.kr (J.H.K.); aowlr8945@gmail.com (Y.U.C.); 2 Department of Materials Science and Engineering, Inha University, Incheon 22212, Republic of Korea

**Keywords:** CoFe_2_O_4_, mica, gas sensor, ethanol, sensing mechanism

## Abstract

In this study, a novel flexible ethanol gas sensor was created by the deposition of a CoFe_2_O_4_ (CFO) thin film on a thin mica substrate using the pulsed laser deposition technique. Transition electron microscopy (TEM) investigations clearly demonstrated the successful growth of CFO on the mica, where a well-defined interface was observed. Ethanol gas-sensing studies showed optimal performance at 200 °C, with the highest response of 19.2 to 100 ppm ethanol. Operating the sensor in self-heating mode under 7 V applied voltage, which corresponds to a temperature of approximately 200 °C, produced a maximal response of 19.2 to 100 ppm ethanol. This aligned with the highest responses observed during testing at 200 °C, confirming the sensor’s accuracy and sensitivity to ethanol under self-heating conditions. In addition, the sensor exhibited good selectivity to ethanol and excellent flexibility, maintaining its high performance after bending and tilting up to 5000 times. As this is the first report on flexible self-heated CFO gas sensors, we believe that this research holds great promise for the future development of high-quality sensors based on this approach.

## 1. Introduction

Ethanol, as an important member of volatile organic compounds (VOCs), is commonly used as a raw material, solvent, and thinner in chemical engineering, agriculture, the pharmaceutical industry, food manufacturing, and clinical and medical applications [1,2,3]. However, it is flammable with an explosion range of 3.3–19 vol% [4]. In addition, excessive inhalation, consumption, or similar exposure to ethanol vapor causes drowsiness, irritation of the eyes, liver damage, breathing difficulties, headache, vertigo, nausea, fatigue, and even anesthesia and damage to the nervous system [5,6,7]. Ethanol consumption by drivers is a major contributor to automotive accidents globally, with impairment and crash risk rising significantly at blood alcohol concentrations over 425 ppm [8]. Thus, detecting ethanol in a driver’s exhaled breath can help identify intoxication and reduce or prevent alcohol-related accidents [9]. Ethanol is also considered a biomarker and measurement of skin ethanol gas can be used to detect human volatile organic chemicals in blood [10]. Ethanol gas sensing thus has important applications for health monitoring and public safety by enabling the non-invasive determination of blood alcohol content and impaired driving. There is a clear need for robust ethanol detection from both an accident prevention and a diagnostic screening perspective.

Resistive gas sensors are among the most prevalent materials for detecting ethanol and other gases because of their high sensitivity, high stability, rapid response time, simple design, simple fabrication, and low cost [11,12]. These sensors are primarily based on semiconducting metal oxides [13]. However, they exhibit high operating temperatures, which lead to high power consumption, poor selectivity, which can lead to false alarms in real applications, and high-humidity interfaces, which can decrease the overall performance of the gas sensor [14]. Accordingly, it is necessary to further explore the sensing properties of the less-studied semiconducting metal oxides to identify new opportunities or to solve existing challenges in the field of resistive gas sensors.

Cobalt ferrite (CoFe_2_O_4_), as a ternary metal oxide, has a spinel crystal structure [15] and good magnetic properties (hard ferrite), mechanical hardness, and chemical stability [16]. Furthermore, it is semiconducting in nature and is therefore used in gas-sensing applications [17,18]. For example, Wei et al. [19] reported synthesis of CFO nanoparticles (NPs) for ethanol sensing, where a response of 110 to 100 ppm ethanol was recorded at 200 °C. In another study, CFO nanorods (NRs) were prepared for acetone sensing at 350 °C, and a response of 57% to 500 ppm acetone was reported [20]. Rathore et al. reported a response of 45% to 200 ppm ethanol at 250 °C [21]. Xiangfeng et al. [22] reported a response of 4 to 10 ppm ethanol at 150 °C. As shown in the examples above, CFO sensors generally operate at relatively high temperatures, which can limit their application in remote areas due to the limited lifetime of batteries and the need to replace them with new ones regularly. Thus, it is necessary to reduce power consumption using different strategies. Operation of the sensor in self-heating mode is a feasible strategy to reduce power consumption through the application of external voltages to the sensor electrodes, where heat is generated via the Joule heating effect. In fact, upon incident of accelerated electrons to other electrons, atoms, and ions, their kinetic energy is lost and part of it is converted to heat [23,24]. 

Furthermore, for new applications, rigid sensors with poor flexibility are not appropriate for universal applications, and sometimes flexibility of the sensor is highly demanded. Nowadays, highly flexible substrates such as paper, plastic, and polymers are widely used to realize flexible gas sensors with high performance owing to their high flexibility, low cost, and high availability. However, they have some shortages and limitations: their thermal stability is not generally high and they can exhibit high thermal expansion, which makes them inappropriate for materials with low thermal expansion coefficients [25,26]. Therefore, in this study, mica sheets were used as flexible substrates because of their high thermal stability, good mechanical flexibility, and low cost [27,28].

Motivated to address the lack of flexible and self-heating CFO gas sensors in the literature; herein, we have developed a highly sensitive ethanol sensor with good flexibility and low-power self-heating mode operation. Initially, CFO thin films were directly deposited onto a mica substrate using the pulsed laser deposition (PLD) technique, which offers the advantages of adaptability, good control over the growth rate, and excellent stoichiometric transfer [29]. After the characterization of CFO thin films, the gas-sensing properties of the fabricated CFO sensor were measured in the presence of ethanol. Under external heating, the sensor performed optimally at 200 °C. However, to reduce power consumption, the sensor was operated in self-heating mode under different applied voltages, showing an enhanced response to ethanol gas at 7 V applied voltage. Furthermore, after bending and tilting for up to 10,000 cycles, the sensor successfully detected ethanol, confirming its high flexibility, which is highly important for new applications.

## 2. Experimental Section

### 2.1. Sample Preparation

Transparent and flexible fluorophlogopite mica KMg_3_(AlSi_3_O_10_)F_2_ (referred to as mica for simplicity) was utilized as the sample substrate. The mica slab was pre-treated with deionized water and mechanically exfoliated to ensure an atomically smooth surface along the (001) direction. Subsequently, a CFO thin film was successfully deposited on the mica surface using the PLD technique, where a Q-switched 4ω Nd:YAG laser (λ = 266 nm, 10 Hz) was focused on the stoichiometric CFO ceramic target, located 5 cm away from the sample surface, with a laser fluence of 0.82 J/cm^2^. During the deposition, the temperature of the mica substrate was fixed at 650 °C, while the oxygen partial pressure was fixed at 0.04 mTorr. The deposited CFO film firmly adhered to the mica without any visible crack or failure. Based on our previous study [30], the deposition rate was fixed at 0.018 nm/pulse, and a layer-by-layer growth method was used to apply the CFO thin film.

### 2.2. Characterizations

The phase and crystalline structures of the films were characterized by high-resolution X-ray diffraction (XRD; D8 Discover, Bruker, Seongnam-si 13493, Republic of Korea) using Cu-*K*α_1_ radiation (λ = 0.15406 nm). The vertical structure of the CFO/mica was investigated by aberration-corrected scanning transmission electron microscopy (AC-STEM; JEM-ARM200F, JEOL, Seoul 05355, Republic of Korea) at a beam energy of 200 kV. For the AC-STEM measurements, cross-sections of the films were prepared using a focused ion beam. Also, the temperature of the gas sensors in self-heating mode was measured at RT with an infrared thermometer (IT-480S, Horiba, Yongin-si 16878, Republic of Korea), and the distance was fixed at 50 mm.

### 2.3. Gas-Sensing Tests

**Figure 1:**
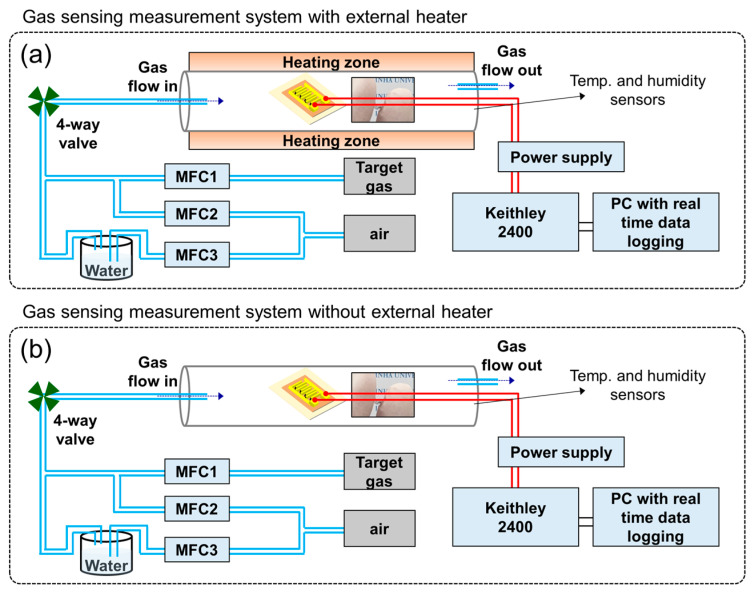
Gas-sensing measurement system (**a**) with and (**b**) without external heater.

To fabricate the gas sensors, a double-layer interdigitated electrode composed of Ti (50 nm) and Pt (200 nm) was sputtered onto the specimens over a mica substrate. The sensing behavior of CFO on mica was studied using C_2_H_5_OH, CH_4_, NH_3_, CO, and C_3_H_5_OH gases. Since acetone and ethanol have almost the same nature, it is important to fabricate a gas sensor to selectively detect ethanol in the presence of acetone. Also, other gases are representative of reducing gases. CO is emitted from fossil fuels, CH_4_ is widely used as fuel, and NH_3_ is widely used in industrial and agricultural processes. Therefore, acetone, CO, CH_4,_ and NH_3_ were selected as interfering gases in this study. Obviously, the selectivity study can be expanded to other gases. However, currently, we have focused on these gases. The gas-sensing tests were performed by electrically connecting the gas sensors to an electrical measuring system (Keithley 2400) interfaced with a computer. During the measurements, the gas sensors were put in a gas chamber (50 × 50 × 1000 mm^3^) in a horizontal-type tube furnace. The target gas concentration was controlled by varying the mixing ratio of the dry air-balanced target gas and dry air (total flow rate = 500 sccm) through mass flow controllers. The resistance of the gas sensor was recorded in the presence of air (R_a_) and target gas (R_g_), and the gas response was calculated as R = R_a_/R_g_. The response and recovery times were defined as the time to reach 90% of the final resistance after exposure to the target gas and the time to recover 90% of the initial resistance of the sensor after removing the target gas, respectively. A schematic diagram of the gas-sensing measuring system is shown in Figure 1. The external heaters of the tubular gas sensor were used to precisely control the sensing temperature (Figure 1a). In addition, in the self-heating mode, the external heater was turned off and, by increasing the applied voltage through the power supply, the temperature of the sensing layer was adjusted (Figure 1b).

## 3. Results and Discussion

### 3.1. Characterization Studies

**Figure 2:**
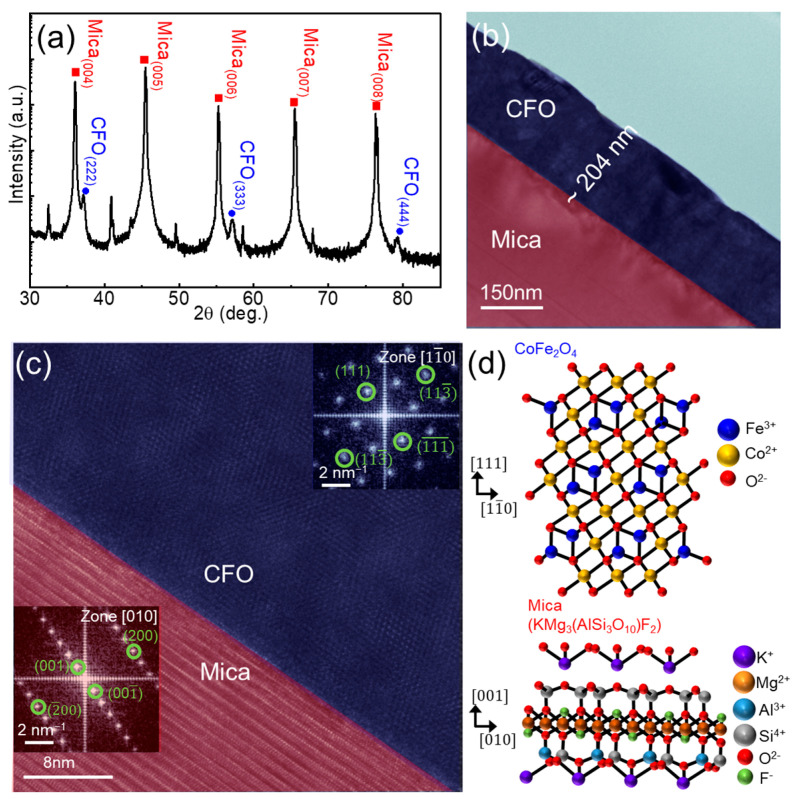
(**a**) XRD pattern of CFO film on a mica substrate. (**b**) Cross-sectional TEM image of CFO on mica substrate. (**c**) False-color TEM image magnified from the interface of (**b**). Insets in each region show the corresponding FFT patterns, revealing the high-quality heteroepitaxy between CFO and mica. (**d**) Schematic illustration of CFO crystal structure formed on a mica substrate via vdW epitaxy.

Figure 2a shows the XRD pattern of CFO on mica, where peaks related to CFO (blue spheres) and mica (red squares) are observed, matching with JCPDS cards No. 22-1086 and 71-1542 for CFO and fluorophlogopite mica, respectively [31,32,33,34]. The high-intensity peaks are associated with Bragg reflections produced by various pairs of mica layers, indicating the maintenance of a high-quality layered mica structure during the PLD deposition. This is attributed to the high melting temperature of mica (~1200 ˚C) [31]. However, the presence of only CFO_(_*_hhh_*_)_ peaks implies the growth of CFO layers on mica, favoring the crystallographic relationship of CFO_[111]_//mica_[001]_. As the cubic structure of CFO (a = 8.394 Å) is different from the monoclinic structure of mica (a = 5.187 Å, b = 9.015 Å, c = 10.131 Å, and β~100°), conventional epitaxial growth using strong chemical interaction is restricted at this exotic hetero-interface. In contrast, van der Waals (vdW) epitaxial (or quasi-vdW epitaxial) growth utilizes weak but long-range vdW interactions rather than the strong chemical bonds observed in conventional epitaxial growth, enabling a larger tolerance of lattice mismatches [35]. Although mica often forms a pseudohexagonal lattice with its matching condition to accommodate the large lattice mismatch, it can be advantageous for forming vdW epitaxy in heterogeneous systems [36,37].

To further investigate the structure of the CFO/mica, a cross-sectional TEM study was performed. The high-resolution TEM image (Figure 2b,c) shows that a CFO film with a thickness of approximately 240 nm was successfully deposited on the mica substrate. There is a clear interface between the CFO and mica, where a highly crystalline CFO film is well synthesized on a flat mica substrate. Additionally, we used fast Fourier transform (FFT) images in the insets, each taken individually from the CFO film and mica substrate near the interface. The zone axes of CFO and mica were calculated as [1$\bar{1}0$] and [010], respectively. The clear dots in the FFT images verify not only the fact that CFO_[111]_ is synthesized on the mica [001] along the vertical direction, but also that CFO [1$\bar{1}0$] is laterally formed along the mica_[010]_ surface, maintaining the epitaxial relation. Moreover, the well-indexed lattice points without any ring patterns or signal blurring in the FFT were consistent with the XRD results, implying the rare presence of defects or granular structures. Based on the XRD and TEM results, a schematic of the growth structure of CFO on the mica substrate was generated, as shown in Figure 2d.

### 3.2. Gas-Sensing Studies

**Figure 3:**
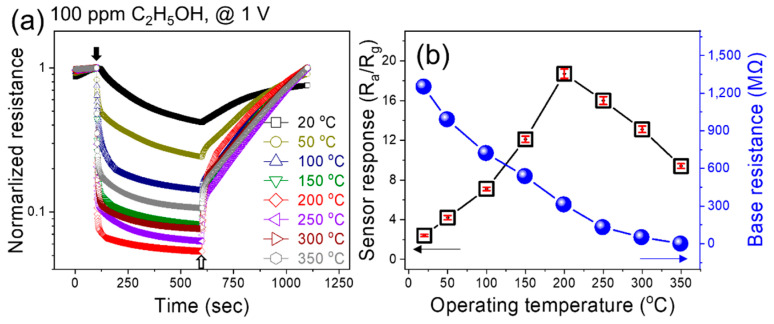
(**a**) Dynamic normalized resistance curves of the CFO sensor to 100 ppm C_2_H_5_OH at various temperatures. (**b**) Corresponding response and base resistance of the sensor versus temperature.

Since the working temperature of the gas sensor directly affects its performance, initially, the fabricated gas sensor was exposed to 100 ppm ethanol at various temperatures from room temperature to 350 °C to find its optimal sensing temperature (Figure 3a). Obviously, the resistance of the sensor decreased upon exposure to ethanol as a reducing gas, reflecting its *n*-type nature. The response was calculated based on variations of the sensor resistance and by increasing the temperature to 200 °C; a response of 19.2 to 100 ppm ethanol was recorded (Figure 3b). A further increase in the sensing temperature led to a decrease in the response owing to the dominancy of the desorption rate of ethanol relative to its adsorption. Also, at lower temperatures, there was not enough energy provided to ethanol vapor to overcome the adsorption barrier. Also, as presented in Figure 3b, with increasing temperature, the resistance continuously decreased owing to the jumping of electrons from the valence band of CFO to the conduction band, resulting in an increase in electrons as major charge carriers, leading to an increase in conductance at higher temperatures.

**Figure 4:**
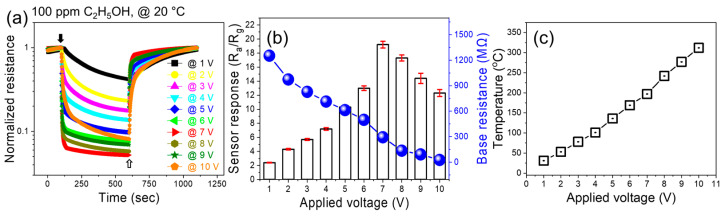
(**a**) Dynamic normalized resistance curves of the CFO sensor to 100 ppm C_2_H_5_OH at various applied voltages. (**b**) Corresponding response and base resistance versus applied voltage. (**c**) Generated temperature versus applied voltage.

Next, the sensor was exposed to 100 ppm ethanol gas under various applied voltages (Figure 4a). The response increased with increasing applied voltage up to a maximum value of 7 V, and then it decreased (Figure 4b). Under 7 V applied voltage, the sensing temperature was approximately 200 °C, which was in accordance with the external heating experiments (Figure 3b). Accordingly, the decrease in the response at higher voltages was due to the generation of a high amount of heat, which increased the sensor temperature beyond 200 °C, where the desorption rate was higher than the adsorption rate. Upon the application of voltage, heat was generated inside the sensor due to a loss of kinetic energy of electrons when they incident with other electrons or ions in their pathways. In addition, increasing the applied voltage causes more electrons to jump into the conduction band, resulting in a decrease in resistance.

**Figure 5:**
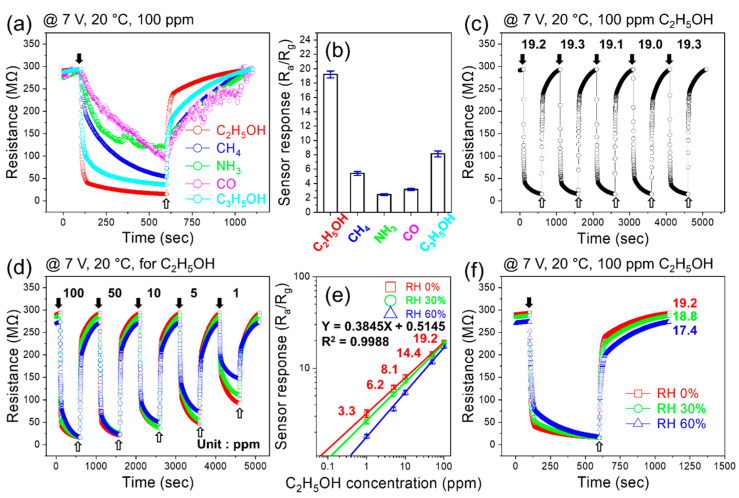
(**a**) Dynamic resistance curves of the sensor for 100 ppm of various gases at 7 V applied voltage and (**b**) corresponding selectivity graph. (**c**) Repeatability of the sensor during five sequential cycles to 100 ppm ethanol at 7 V applied voltage. (**d**) Dynamic resistance curve and (**e**) corresponding calibration curve for low concentrations of ethanol at 7 V applied voltage. (**f**) Dynamic resistance curves for 100 ppm ethanol at 7 V applied voltage under 0, 30, and 60% RH.

For practical applications, the sensor should display good selectivity for ethanol gas; otherwise, false alarms lead to the wrong detection of gas. Therefore, to study the selectivity of the sensor, it was exposed to various toxic gases. Figure 5a shows the dynamic resistance curves of the sensor to 100 ppm of various gases at a fixed applied voltage of 7 V, and Figure 5b shows the corresponding selectivity graph of the sensor. The response values to 100 ppm ethanol, methane (CH_4_), ammonia (NH_3_), carbon monoxide (CO), and acetone (C_3_H_6_O) gases were 19.2, 5.4, 2.5, 3.2, and 8.1, respectively. Thus, the sensor exhibited good selectivity for ethanol gas. Furthermore, the response to ethanol was almost three times higher than the response to acetone. Figure 5c shows the repeatability of the sensor’s performance during five sequential cycles to 100 ppm ethanol under an applied voltage of 7 V. The response values during the first, second, third, fourth, and fifth cycles were 19.2, 19.3, 19.1, 19, and 19.3, respectively, indicating good repeatability, which is important from an application perspective. We also examined the response of the sensor to lower ethanol concentrations at a fixed applied voltage of 7 V (Figure 5d). Corresponding calibration curves (Figure 5e) clearly confirm that the sensor is able to detect ethanol gas. Figure 5d gives the response of the optimized sensor to 1, 5, 10, 50, and 100 ppm C_2_H_5_OH at 7 V, and Figure 5e displays the variations of the sensor response on the ppm concentrations of C_2_H_5_OH. We estimated the detection limit (LOD) of the gas sensor (sensor response = 1.1) by extrapolation using a log–log scale plot. The LOD was 70 ppb, demonstrating the high potential of the sensor for C_2_H_5_OH gas detection. Also, by the same procedure, LOD in the presence of 30 and 60% RH were 120 and 400 ppb, respectively. In addition, the response of the sensor in the presence of various levels of humid air with relative humidity (RH) levels of 0, 30, and 60% was studied at an applied voltage of 7 V (Figure 5f). The response in dry air without humidity was 19.2, which decreased to 18.4 and 17.4 at 30 and 60% RH, respectively. Table 1 shows the response, response time, and recovery time to 100 ppm ethanol in the presence of various levels of RH. Overall, all three parameters become worse with increasing humidity levels. In fact, not only does the response decrease, but its response time and recovery time also become longer. Although the response decreased in the presence of humidity, it was still high enough for practical applications. In humid environments, water molecules occupy the adsorption sites, decreasing the available adsorption sites for gas molecules. Thus, a smaller amount of ethanol could be adsorbed onto the sensor, resulting in a lower response than under dry conditions. In addition, the baseline resistance increases under humid conditions owing to the dissociation of H_2_O molecules from the sensor and the generation of electrons in the *n*-type sensing layer, thereby increasing the base resistance of the sensor [38,39,40].

**Figure 6:**
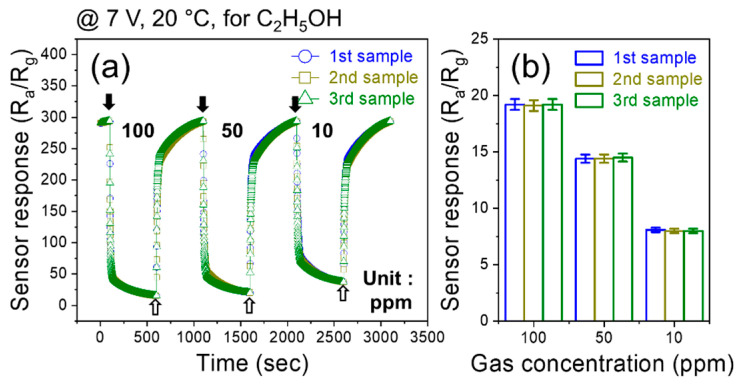
(**a**) Dynamic resistance curves of three fabricated sensors to various concentrations of C_2_H_5_OH at 7 V applied voltage and (**b**) corresponding selectivity graph.

To check the reproducibility of the sensor, we fabricated three gas sensors using the same procedure and the same materials. Then, they were exposed to 10, 50, and 100 ppm C_2_H_5_OH gas at a fixed 7 V applied voltage (Figure 6a). All three fabricated gas sensors showed the same sensing behavior, where their resistances decreased to the same value upon exposure to the same gas concentration. Also, in Figure 6b, the response versus gas concentration is depicted for three gas sensors, where all fabricated gas sensors show the same response to gas. This clearly demonstrates the high reproducibility of the gas sensor in this study.

In Table 2, we compare the ethanol gas-sensing performance of the present sensor with those reported in the literature. The present sensor shows a response of 19.2 to 100 ppm ethanol under 7 V applied voltage, which is an acceptable performance considering the sensing temperature and the gas response of other sensors. Even though some sensors in Table 2 have higher sensing performance than the present sensor, their operation temperature is significantly higher. For example, temperatures up to 300 °C have been reported for ethanol sensing, which lead to significant power consumption in real applications.

**Figure 7:**
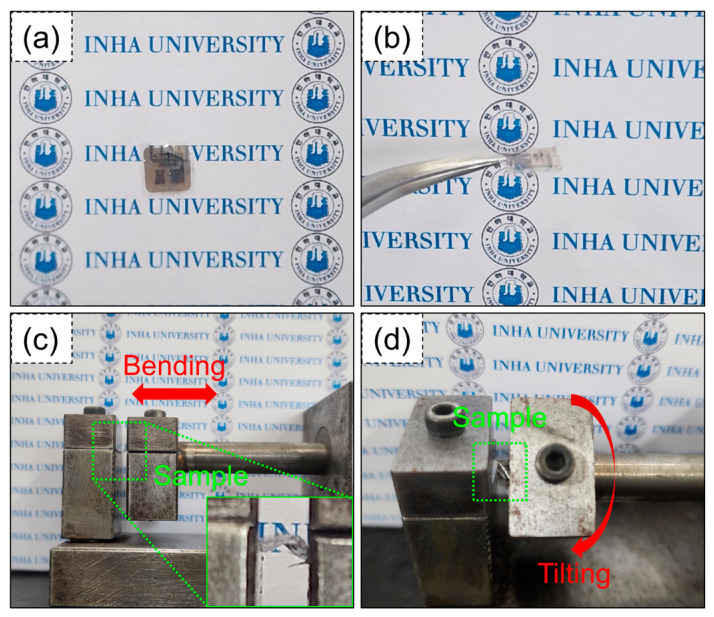
(**a**,**b**) Digital photographs of fabricated flexible gas sensor apparatus for (**c**) bending and (**d**) tilting of the flexible sensor. Inset in (**c**) shows higher magnification image.

Next, we measured the sensing properties under flexible conditions. Digital photographs of the fabricated flexible gas sensors are presented in Figure 7a,b. In addition to flexibility, the sensor exhibits good transparency, which is important for some applications. Figure 7c,d display the apparatus and conditions for bending and tilting the flexible sensors. The inset in Figure 7c shows an image of the sensor when bent.

**Figure 8:**
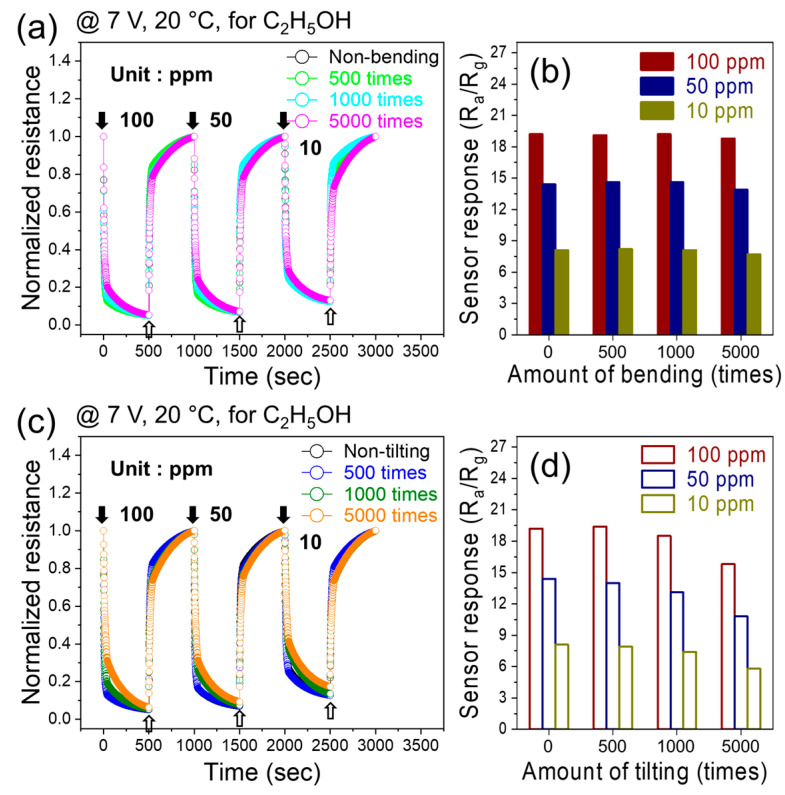
Dynamic resistance curves of flexible sensor for 10, 50, and 100 ppm ethanol gas at 7 V applied voltage after various (**a**) bending and (**c**) tilting cycles. Corresponding response versus (**b**) bending and (**d**) tilting cycles.

The dynamic resistance curves of the flexible sensor for 10, 50, and 100 ppm of ethanol gas at an applied voltage of 7 V after various numbers of bending cycles (500, 1000, and 5000 cycles) are shown in Figure 8a. The response values were calculated, and Figure 8b shows that the response did not change significantly in response to varying numbers of bending cycles. This demonstrates the high flexibility of the fabricated gas sensors. We also tested the sensing behavior after tilting the sensor 500, 1000, and 5000 times with various concentrations of ethanol at a fixed applied voltage of 7 V (Figure 8c). The response shows no significant changes in performance after these repeated tilting cycles, which again confirmed the high flexibility of the CFO thin film sensor (Figure 8d).

### 3.3. Gas-Sensing Mechanism

The sensor in this study exhibited *n*-type-sensing behavior, indicating that the sensing mechanism relies on the formation of an electron depletion layer (EDL) on the sensor surface, as is commonly accepted for *n*-type gas sensors. When the sensor is exposed to air, oxygen molecules with high electron affinity are adsorbed on the sensor surface, and electrons are extracted. The relevant reactions are as follows [47].

(1) $$O_{2}(g)\to O_{2}(ads)$$

(2) $$O_{2}\left(ads\right)+e^{-}\to O_{2}^{-}\left(ads\right),\;T\leq 150\;{}^{\circ}C$$

(3) $$O_{2}^{-}\left(ads\right)+e^{-}\to {2O}^{-},\;150\;{}^{\circ}C\leq T\leq 400\;{}^{\circ}C$$

(4) $$O^{-}+e^{-}\to O^{2-},\;T>400\;{}^{\circ}C$$

Thus, an EDL was formed on the surface of the sensor, and the concentration of electrons in this layer was lower than that in the core. Thus, the resistance of the sensor was higher relative to vacuum conditions, where there was no oxygen. Upon exposure to ethanol, the following reaction is believed to occur at the sensing temperature [48].

(5) $$C_{2}H_{5}OH+{7O}^{-}\to 2CO_{2}+{3H}_{2}O+7e^{-}$$

Therefore, the reaction of the adsorbed oxygen species with the ethanol molecules leads electrons to be released onto the sensor surface. Thus, the thickness of the EDL and the resistance decreased. This results in the appearance of a sensing signal. Furthermore, in the contact areas between the CFO grains, double Schottky barriers initially form in air, leading to difficulty in the movement of electrons across the grains, and upon exposure to ethanol vapor, the height of these potential barriers change, resulting in a change in the sensor resistance. Accordingly, a part of the generated sensing signal is due to the contact area between CFO grains.

## 4. Conclusions

In brief, using the PLD deposition technique, a CFO thin film was successfully deposited on a thin mica substrate using a solid CFO target to create a flexible ethanol sensor. AC-STEM analysis confirmed that CFO was successfully deposited on the substrate with a well-defined interface. At 200 °C, the fabricated sensor exhibited the highest response of 19.2 to 100 ppm ethanol. To reduce power consumption, the sensor was operated in self-heating mode, where an applied voltage of 7 V resulted in a high sensor response of 19.2. The sensor was able to detect as little as 1 ppm of ethanol, and its response did not significantly decrease in humid air. The sensor also demonstrated high flexibility, and its performance did not degrade after bending and tilting up to 5000 times. Thus, this study realized the creation of a novel flexible, self-heating, selective, and sensitive ethanol sensor based on CFO on a mica substrate. The results presented in this manuscript pave the way for future studies in this area.

## Figures and Tables

**Table 1:** Response, response time, and recovery time of the sensor in the presence of various levels of RH.

RH (%)	Response (R_a_/R_g_)	Reaction Time (s)	Recovery Time (s)
0	19.2	35	170
30	18.8	92	202
60	17.4	194	237

**Table 2:** Comparison of ethanol-sensing performance of present sensor with those reported in the literature.

Sensing Materials	Conc. (ppm)	T (°C)	Response (R_a_/R_g_ or R_g_/R_a_)	Ref.
CFO-400	100	200	110	[19]
CoFe_2_O_4_ nano-crystallines	1000	150	71.9	[22]
ZnFe_2_O_4_ nanoparticles assembled nanosheets/RGO	100	210	41.5	[41]
CdFe_2_O_4_ calcined at 700 °C	100	250	55	[42]
ZnO nanoparticles	100	180	121.5	[43]
ZnO/Co_3_O_4_ nanostructures	10	300	34.9	[44]
NiO/ZnO	100	300	5.4	[45]
ZnO@In_2_O_3_ core@shell nanofibers	100	225	31.87	[46]
CFO on a flexible mica substrate (this work)	100	20 (7 V)	19.2	This work

## Data Availability

Data will be made available on request.
